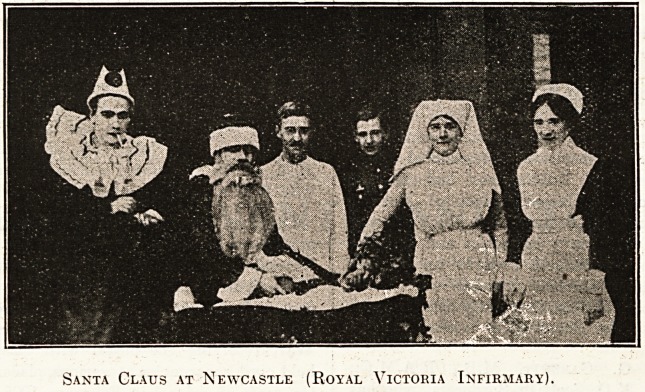# Some Military and Provincial Hospitals

**Published:** 1916-01-01

**Authors:** 


### SOME MILITARY AND PROVINCIAL HOSPITALS.

### County of London War Hospital, Epsom.

### THE AMUSEMENTS.

On Christmas Eve a most successful concert was given in the large theatre (which will seat 2,000) by the lady almoner (Right Hon. Lady St. Helier). Mr. Robinson and other entertainers delighted for nearly two hours those who were well enough to be present. Outside the hospital the church choirs from the neighbourhood sang carols to those gallant comrades too ill to be otherwise than in bed. Christmas morning the fun commenced. Quite early, Christmas hymns could be heard, and soon bands of the more convalescent Tommies, in fancy-dress costumes, were conveying greetings everywhere, chiefly in song, accompanied by such musical instruments as are commonly found in Christmas stockings. Each soldier found in his locker a Christmas card

**Wishing you a Merry Xmas and a Happy New Year.**

The Entrance Block.

specially designed and printed by the hospital. At 11 a.m. those so inclined went to church, and quite a large number of both staff and patients attended early communion. The work of the hospital could not stop even at Christmas, but all the soldiers were keen upon being free for the afternoon and evening.

After the medical visit and inspection the day was given over to seasonable amusements of a real old-fashioned Christmas character. Christmas dinner consisted of beef, pork (and apple sauce), plum-pudding, dessert, etc., and was a great success.

The officer commanding had relaxed the standing order against inter-ward visiting, and soon parties of patients and staff were interchanging happy greetings. At 4.15 a representative of the lady almoner gave out the presents provided by her department and Colonial authorities, Christmas-trees were dismantled, and after tea song and games became universal, except in the vicinity of those very ill. The tea was a great function. Each ward had a large decorated Christmas cake and a good supply of Christmas crackers, and in most wards a specially arranged programme of music was carried out.

The officer commanding (Lieut.-Colonel J. R. Lord) paid a visit to every ward, spoke to all those in bed. The London County Council was represented by the chairman of the hospital, Mr. H. J. Greenwood, who visited everywhere, comforting, consoling, cheering, and thanking as is his wont.

At present there are a large number of Australian, New Zealand, and Canadian patients in the hospital, who passed a really happy day—one they will always remember, and carry the recollection of their first Christmas in England to those distant lands they are so proud of. They were made to feel thoroughly at home, and did not hesitate to say so. Greetings were sent them, also Christmas cards, by Colonial authorities and organisations.

### THE FLORAL DECORATIONS.

High praise is due to the sisters and nursing staff of all wards in this hospital, who, with the available assistance of patients, have gone out of their way with untiring energy, and in some cases incurred considerable expense, in order to provide the boys with every possible solace and enjoyment, with the result that infinitely pretty scenes meet the eye. The art of floral decoration has been thoroughly put into practice, and no stone has been left unturned to maintain the good old customs consistent with the occasion.

If an established floral artist had been chartered for the purpose of designing the decorations in the first ward which we visit, a big medical ward, the result could not have possibly been more effectively satisfactory than the array of beauty displayed. The originality, and at the same time almost perfect reality of the hand-made floral decorations, disposed to their best advantage, makes it an almost easy matter to imagine they are Nature's own produce. Consistent with the occasion is the proverbial Christmas-tree, laden with dainties, etc., and lighted by electricity, which, with its almost sentimental

*L.N.A.]* *[Photo.*

Toys for the Wounded. The Matron of the 3rd London General Hospital with an Armful of Toys.

reminder, goes to show how nothing has been spared to ensure the lads a good old-fashioned Christmas.

In another ward the abundance of evergreen bowers and holly suggests that no time and care have been wasted in order to make the ward as outstanding as possible. And, indeed, the workers here have done so, and they have made a novel feature by placing oranges amongst the evergreens, so that with Chinese lanterns hung here and there the result is very effective. We then come to a ward with perhaps not so varied and extensive decorations, yet a dainty and neat adornment of evergreens and paper roses. If the nature of the ward does not permit of a really effective display the workers have at any rate surmounted that difficulty by making it look as bright and cheery as is possible under the circumstances.

Another ward presents a delightful scene, with windows adorned with evergreens, electric globes decorated with holly, and mantelshelves arrayed with imitation roses, clematis, almond blossom, and tulips. The clematis is especially worthy of praise. There is a huge snow-pie (made with wool) containing presents for patients, and draped with the Union Jack, and the Christmas-tree has not been forgotten. Then as we leave we find the corridor arrayed with bunting and evergreens, and on a window, humorous in its crudeness, is the greeting, " 'Ome sweet 'Ome." One ward has a strikingly pretty floral painted dado around the walls, and around the windows are arrayed flags of all nations, and there are garlands of evergreens and holly. An enterprising member of this ward has instituted a Christmas programme which is posted up, and includes games, concerts, competitions, etc., for the whole Christmas week. They intend not to be dull here.

As we go from ward to ward we find scenes similarly charming in their variety of decoration. One ward in particular has a very significant and novel feature in having draped the pillars with muslin in patriotic colours of the Allies, which are really worthy of praise. Affixed to the Australian pillar are the national emblems, the Emu and the Kangaroo. At the same time this ward has not concentrated its main efforts on the pillars, because the whole room is decked with evergreen chains, with holly garlands round windows and doors. And there are hanging baskets of various colours containing pretty evergreens, and across the ward on a muslin band are engrossed the greetings of the season.

In two busy surgical wards one is almost surprised to observe that in a comparatively short space of time a charming transformation has been effected, and inasmuch as in both instances they contain a large number of critical surgical cases, and at the same time very few able patients, great credit is reflected on the staff for having produced pretty scenes with the usual decorative material, but an absolutely harmonious principle, so that even with a moderate display everything has been made to appear cheery and inviting.

### 3rd London General Hospital, Wandsworth, S.W.

To describe Christmas at the 3rd London General is no easy task, partly because the festivities were so multitudinous in their detail, but mainly because what really mattered was the atmosphere—the something intangible and inexpressible which we call the "spirit" of the season. That spirit pervaded the whole of this great building (or, rather, conglomeration of buildings), and must have been felt to be understood. Mere words fail to define it.

There are many Anzac patients here, and perhaps their enthusiasm is the best tribute. For they were, one and all, curious to learn what "an English Christmas" was like. And they are keen critics of the homeland and its ways. But the English Christmas was no disappointment. Its spontaneous jollity exceeded their expectations. It is nice to think, too, that the lavishness of the gifts which were showered upon all in the hospital must have given the Anzacs a pleasant impression of just what the British public thinks of the soldiers who have fought for the country. Indeed, the endless stream of packages of every sort—crates, baskets, parcels, bundles—which flowed in at the hospital's main portal on the 24th was a touching testimonial to the love and pride with which our wounded heroes are regarded not only by their personal acquaintances and relatives, but also by a host of unknown people. The matron and her staff had a severe task in allotting all the good things which arrived; they laboured long and late to divert the stream of gifts into the proper channels. But the present writer would be surprised to hear that any recipient was disappointed or that any of the vast store of dainties and other presents failed to arrive just where it ought.

All the wards, of course, were decorated, many with a good deal of originality, though anything novel in Christmas decoration is no easy feat to compass. In some cases much of the ornamental material—artificial flowers—had been made by the wounded themselves. One ward, by means of flakes of cotton-wool fixed to invisible threads, represented Winter, with a snow-storm falling. Its neighbour was Summer, bowered in blossoms made by the patients. Holly and mistletoe, needless to say, was to be found everywhere.

On Christmas Eve, after dark, a charming procession of sisters, nurses, and probationers paraded the corridors and wards, with Chinese lanterns, singing carols. On Christmas morning every patient woke up to find a stocking awaiting him and filled with good things by the wife and daughters of Lieut.-Colonel Bruce Porter (O.C. of the hospital); also, of course, other presents as well. The event of the day was the dinner in the wards, at which, in each case, the ward staff sat down with the patients and a very merry "family" tone of comradeship prevailed. In one of the wards Queen Amélie of Portugal, who is a nurse here, ate her Christmas dinner with her patients. Meanwhile the C.O. and Mrs. Bruce Porter and their daughters, with the matron (Miss Holden), were making their round of the hospital. Every patient and member of the staff was personally shaken hands with, and each patient received a buttonhole of flowers appropriate to his nationality (*e.g.*, the Anzacs received wattle). In every ward the party exchanged a few words with the assemblage and there were enthusiastic cheers for the C.O. and matron. This ceremonial took place during the dinner hour, and as the hospital contains upwards of sixty wards the peregrination must have been "some" job for the Colonel and his party.

All the members of the staff, however, seemed tireless on Christmas Day. Dinner, and the merrymakings which followed it, were hardly over before a colossal tea-party began in the receiving hall, where all walking patients were entertained and where an orchestra played from the gallery. Immediately afterwards a professional company of stage entertainers gave a capital concert, which was packed; the neighbouring recreation room also was crowded for a moving-picture show. Both were visited by Princess Louise (Duchess of Argyll), who had come down for the tea-party, and who had brought a special message from the King to the 3rd London General Hospital patients.

In the evening the orderlies had their Christmas dinner. Merrymaking in the orderlies' quarters was kept up until a late hour, their own small orchestra providing music for a smoking concert. On the following evening the sisters and officers held their banquet.

One small feature of the Christmas festivities is worth recording in conclusion, as showing the minuteness of detail with which everything had been thought out. Every patient and every member of the staff received, *personally addressed to himself or herself*, a Christmas card from the Colonel; no ordinary card, for it had been specially made—designed, under the C.O.'s careful direction, by one of the orderlies (Private Irving, a professional artist), and enshrining "The Monk's Vow," a motto which the Colonel always keeps framed in his own office. The wording, than which none could have been more beautiful and appropriate for both staff and patients of an institution such as this, is as follows:

### THE MONK'S VOW.

"I shall pass through this world but once: Any good thing therefore I can do, or any kindness that I can show to any human being, let me do it now; let me not defer it or neglect it, for I shall not pass this way again."

A wonderful theatrical entertainment was given by the staff to the patients of the hospital and to the children of the Royal Patriotic School on December 27, 29, and 30. It took place in the receiving hall and was performed to a packed and appreciative audience of patients and staff, amongst other guests present at the first performance being Colonel Peterkin, D.D.M.S. The programme included Gilbert and Sullivan's "Trial by Jury," which was produced and conducted by Corporal Craft, R.A.M.C., the artistes being Lieut. L. L. Preston, who took the part of the judge; Miss Melville, V.A.D., the plaintiff; Private Hilliard, R.A.M.C., defendant; Private Kemp, R.A.M.C., counsel for the plaintiff; Private R. Smith, R.A.M.C., Lieut. Beven, Private Scannell, R.A.M.C., Miss Thornton, V.A.D., and Private Vidal, R.A.M.C. (accompanist). This was followed by "The Grey Parrot," acted by Sergeants W. T. Evans and R. R. Hewitson, R.A.M.C., Lance-Corporal F. W. Goodden, R.A.M.C., Staff-Sergeant L. G. Godden, R.A.M.C. (who also produced the sketch), and the Misses F. Boyd and M. Gribble, T.F.N.S. The concluding item was "A Sister to Assist 'Er," performed by Private Hilliard, R.A.M.C., and Miss Walker, T.F.N.S. During the intervals the 3rd London General Hospital orchestra played a selection of music, whilst songs, duets, violin solos, dances, riddles, and recitations were contributed by the 3rd London General Hospital Pierrot Troupe. The entertainment is to be repeated to-day (Saturday).

At the 3rd London General Hospital, Wandsworth. Distributing the Queen's Gifts.

### Out of the Limelight at Winchmore Hill.

### CHRISTMAS AT AN M.A.B. HOSPITAL.

We haven't any soldiers at the Northern Hospital, and, as most of our patients are convalescent fever cases, we are rather out of the limelight. But for some time past seven of our pavilions have been given over to tubercular cases, and so a new element has been introduced into our Christmas festivities. The Northern Hospital is not, strictly speaking, a single hospital, but a collection of over twenty small hospitals. The pavilions are mostly two-storied buildings, and all disconnected from one another. The patients in these pavilions are also kept separate, so that even at Christmas time there can be no joyful congregation of our patients. As usual, this year practically every fever pavilion had its Christmas-tree, all brilliantly decorated, for our patients are nearly all little ones; only a sprinkling of bigger girls and a few adult females is the normal proportion. The dinners this year did not consist of the usual turkey, but the patients had to be content with juicy ribs of beef. But this was the only alteration, for plum-puddings, sweets, crackers, etc., were as plentiful as of yore. In each pavilion, after dinner, high revelry proceeded on independent though customary lines, with music and games for the children. In the pavilions which sheltered the adult tuberculosis patients some degree of mixing was permitted after the feast of the day, a fair proportion of the two hundred consumptives were able to witness a playlet which was very creditably performed in one of the wards by a composite company of nurses and patients. The absence of visitors on such a day would forcibly strike anyone accustomed to the scenes so general in large London hospitals, but the reason is obvious; even the tubercular patients are allowed visitors only on alternate Sundays. The fun permitted to these latter cases has to be somewhat restricted, but as the more severely ill are housed, or rather, bedded, in pavilions by themselves, those in the "sanatorium" blocks are mostly capable of enjoying a certain amount of excitement and exercise without harm accruing. As for the staff, which, as may be imagined, is a large one, their turns came in sections. The doctors had their spread on Christmas Eve, the nurses on Boxing Day, and the sisters' party took place on Tuesday evening. The war rations ran this year to turkeys, but it is said that the puddings had a few eggs less than usual! The customary dance took place on Saturday evening, and was enjoyed by nurses, maids, and porters alike. In short, at the Northern Hospital the holiday interlude was enjoyed by everyone, and our indefatigable matron, Miss Morgan, managed to overcome all warlike difficulties, and to give her large family of nurses, as well as the hundreds of patients, a thoroughly good time.

### National Sanatorium, Benenden.

The National Sanatorium, Benenden, is away from all the world in the heart of the rural scenery of Kent. From the front terrace of the sanatorium one can see many miles of undulating fields dotted with windmills and oast-houses. On Christmas Day the terrace was swept by the wind and the rain, but the dreariness of the outlook heightened the brightness of the interior, and one and all had a happy day, despite the weather.

The patients had the usual Christmas fare—turkey, plum-pudding, mince-pies, trifles, and other delicacies, which were laid out on a long table in the dining-hall, where all dined together. The dining-hall was artistically decorated with holly, laurels, and rich bunting. The table was laid in a very elegant fashion, and increased in colour by the crackers which provided the patients with weird forms of headgear and added to their mirth. Cigars and cigarettes were supplied in abundance.

After a very good tea the evening entertainment began. This was also held in the dining-hall, where a stage had been erected by the carpenter. The footlights consisted of a row of candles, and although the arrangements were somewhat amateurish, the stage when finished looked quite pretty lit up by candlelight.

The programme consisted of a concert given by various members of the staff and the patients themselves, and a dramatic sketch given by the nurses. The audience consisted of the patients and staff, who showed their appreciation of the talent of the actresses and singers by frequent expressions of applause. In this pleasant manner a happy evening came all too soon to its ending.

### Bradford.

Although shorn of some of the outstanding features of previous years, Christmas at the Bradford Royal Infirmary was kept with a degree of heartiness well befitting the great anniversary. The wards were decorated with palms, ferns, and evergreens, each ward sister had shown great taste, and had put in a large amount of work in order to make the wards present a gay and bright appearance to the patients and their friends. Each patient in the hospital received a suitable present, which was given to them by Father Christmas himself in the person of the resident surgical officer.

The infirmary was visited on Christmas morning by the Lord Mayor and Lady Mayoress of the city, accompanied by many prominent citizens, and a tour was made of the wards of the hospital. The Christmas dinner, which was provided by the Lord Mayor, was greatly enjoyed; each patient who was sufficiently well received a portion of turkey and plum pudding, and afterwards the men enjoyed their smoke from pipes especially provided for the occasion.

In the course of a speech thanking the Lord Mayor for attending the hospital and providing the Christmas dinner, the chairman of the house committee (Mr. George Priestman) referred to the many members of the infirmary staff who were now serving their country in the Navy and Army, and to the large amount of work which devolved on the officers who remained.

The other charitable institutions of the city were afterwards visited by the Lord Mayor and Lady Mayoress and their party, who were warmly welcomed.

At the Royal Eye and Ear Hospital reference was made by Dr. Campbell, in seconding a vote of thanks to the Lord Mayor, to the new infirmary scheme at Daisy Hill. Dr. Campbell expressed the opinion that the time was now opportune for making a renewed effort to carry forward the scheme, and hoped that when the time came to commence building some plan would be formulated to unite the medical charities of the city in the new building, thus securing greater unity and more economical management.

### Addenbrooke's Hospital, Cambridge.

### HOW CHRISTMAS WAS PREPARED FOR AND HOW IT WAS SPENT.

The preparations for keeping Christmas at Addenbrooke's differed this year from that of past years inasmuch as in addition to the large number of civil patients in the hospital there were about 100 wounded soldiers and a larger staff; thus provision had to be made for about 360 persons under entirely new conditions.

Conscious of the eagle-eyed critic, care has for some years been taken to give no opportunity for complaining that the general funds of the hospital are being used or appropriated for providing festivities (which are so often looked upon as, or in the nature of luxuries) by organising a special fund. Preparations for Christmas began early in November by the making of the time-honoured plum puddings, but owing to the abnormal circumstances they were fewer in number. The secretary-superintendent (Mr. Richard J. Coles), who established the custom some eleven years since, commenced his annual appeal for contributions. The special appeal was made, as usual, firstly through the columns of the local newspapers by letters to the editors, and secondly by writing special letters to a large number of friends of the hospital soliciting their sympathy and support. As a result the contributions in the form of money received in response to the appeal are substantially in excess of those received in any previous year.

This year the sisters and nurses organised a Patriotic Troupe to entertain the patients at Christmastide, for weeks devoting their leisure moments to attaining efficiency in order that they might give of their very best for the enjoyment of the patients. Those who took part in the troupe were dressed to represent the Allied nations and Colonies—viz. : Britannia, Miss Tilley, sister of Griffith Ward; Ireland, Nurse Buckingham; Scotland, Nurse Cotes; Wales, Nurse E. Smith; France, Nurse Emly; Belgium, Nurse Devette; Italy, Nurse Foster; Serbia, Nurse Pinck; Russia, Nurse Lamplough; Australia, Nurse Jennings; Canada, Nurse Peel; India, Miss Sendall, sister Hatton Ward; Egypt, Nurse Johnson; Japan, Nurse Ball; British Lion, Nurse Lane; (England) Huntsman, Nurse Heaton Ellis; Clown, Nurse Simons.

#### Decorations.

For several days before Christmas all were astir, and the sisters and nurses and patients and wounded soldiers who were able were very busy with the decorations, and by the Eve of Christmas the wards throughout the hospital were decorated and illuminated.

In the Bowker Ward the sister, Miss Taylor, with the assistance of her nurses and some of the wounded soldiers, employed with great taste rustic arches, palms, choice plants and flowers, festoons of evergreens, very pretty lamp-shades. The bed-pull brackets were bound with ivy, and Japanese lanterns suspended therefrom. The tables were decorated with choice flowers and fairy-lights.

In the Griffith Ward Miss Tilley, the sister, and her staff of nurses, with the assistance of some of the wounded decorated with great taste almost entirely by means of flags of the Allied nations. The flags were artistically displayed on the walls at the end of the ward, the columns and streamers from one side of the ward to the other. There were plants and evergreens. Tables were decorated with flowers, artistic candlesticks and shades, and fairy-lamps. The general effect of this ward was very pretty and surpassed the decorations of past years.

In the Albert Ward Miss Bird, the sister, and her nurses, again with the assistance of some of the wounded soldiers, decorated with evergreens, plants, choice flowers, etc. The electric lamps were covered with fairy shades. The bed-pulls in this ward also were entwined with evergreens, and Chinese lanterns hung therefrom, and there was a select display of fairy-lamps.

The Tipperary Ward was effectively decorated by the sister, Miss Langresh, and her staff of nurses. On the walls on either side of the ward were suspended festoons in green and pink paper. The entrance to this ward was bedecked with evergreens, and an antique lantern was suspended from the centre. Miss Ormrod, the sister, and her nurses decorated the Victoria Ward with evergreens, plants, Chinese lanterns, and fairy lamps, giving a very pleasing effect. The tables here again were decorated with choice flowers, special candlesticks, and fairy-lamps. The Hatton Ward was decorated by the sister, Miss Sendall, and her nurses with evergreens, fancy-paper admiral caps, bows, etc., suspended from the bed-pulls. There were also plants, Japanese lanterns, etc. Indeed, the whole of the decorations throughout the hospital reflects the greatest credit upon the sisters, nurses, and those patients who were able to assist, the general effect being far superior to that of other years.

A GAILY DECORATED WARD.
THE ROYAL VICTORIA INFIRMARY, NEWCASTLE-ON-TYNE.

On Christmas Eve the matron, sisters, and nurses, carrying Chinese lanterns, proceeded through all the wards singing carols. On the Thursday preceding Christmas Day Mrs. Clay gave a Bethlehem tableau in the Griffith Ward. This was a new departure, proved instructive, and was much appreciated.

On Christmas morning the patients were very busy unpacking their parcels of Christmas gifts. At 12 o'clock dinner, consisting of roast turkey, sausages, potatoes, Brussels sprouts, plum-pudding, mince-pies, dessert, and crackers, was served in all the wards. Each ward had its own special carver: Hatton Ward, Mr. Fitzgerald; Tipperary Ward, Lieut. Bonser, R.A.M.C. (T.); Albert Ward, Captain Searle, R.A.M.C. (T.); Griffith Ward, Captain Budd, R.A.M.C. (T.); Victoria Ward, Lieut. Anson, R.N.; Bowker Ward and Isolation Ward, the Secretary Superintendent; Mr. Richard J. Coles dressed as John Bull. At 12.30 the domestic staff had dinner.

At 3 P.M. and onwards the Patriotic Troupe performed in each of the wards in turn, and were greatly appreciated and applauded.

The sisters and nurses this year, instead of giving presents to one another, sent twenty-four parcels of useful gifts to prisoners of war in Germany.

"A Crib" was arranged in a spare room in the old out-patient department.

### 1st Eastern General Hospital, Cambridge.

THE wounded soldiers at the Military Hospital on King's and Clare Playing Fields, Cambridge, had a very enjoyable Christmas. All the wards were gaily decorated. The festivities commenced at 5.30 on Christmas Eve with a series of beautiful tableaux of the Nativity, arranged by Mrs. Clay and the Vicar of St. Giles', given in the recreation room by Mrs. Bouquet, Miss Clay, the Revs. Ostrehan, Howe, Smith, and Carpenter, and other friends. The tableaux were interspersed with carols and hymns by St. Giles' choir boys. At 7 P.M. the lights were all turned down, and a party, arranged by Major Roderick, and composed of members of the nursing staff and orderlies, assisted by a few voices from outside, went round the wards carrying Chinese lanterns, and sang carols, which were greatly enjoyed by the patients.

The hospital was early astir on Christmas morning. Many of the men had hung up their stockings and their expectations of receiving something from Santa Claus were not disappointed. The rifling of the stockings commenced as early as four o'clock, to the accompaniment of much laughter, whistling, and singing. Some of the soldiers were at their devotions as early as 6.30, when there was celebration of the Holy Communion in the chapel. Later there was Mass for the Roman Catholics, and at 9.30 there was a general church parade, the service being held in the recreation room. This was followed at 10.30 by another celebration of the Holy Communion. The services were conducted by the chaplains, the Revs. Chase and Simpson, and Monsignor Barnes.

The next great event of the day was the distribution of Christmas gifts, which took place about eleven o'clock.

Messrs. Cadbury Bros. kindly sent a large box of chocolate for each patient and member of the staff, and a well-known Cambridge tradesman sent a box of cigarettes and packet of tobacco for each man. Special presents for the Australians were received from the Australian Red Cross Society, and for the Irishmen from the Rangoon Fund for Irish Soldiers. A very welcome consignment of fruit was also received from the *Brisbane Courier* Fund, Queensland. The gifts were distributed by the wives, children, and friends of the medical staff. One of the soldiers dressed up as a wizard also went the round of the hospital distributing fruit and causing much fun and laughter.

At 12 noon dinner was served. This consisted of turkeys and sausages, vegetables, plum-puddings, mince-pies, and fruit. Among the carvers were the Lord-Lieutenant (Mr. C. R. W. Adeane), who officiated in Ward 22. Mrs. Adeane and Miss Lettice Adeane kindly helped to serve the men, and presented dessert and a carnation to each man in the ward. The carvers in the other wards were the ward medical officers, assisted by Messrs. R. Grant, T. Parr, W. Jones, and W. Newman. An offer of Christmas puddings was received from the London *Daily News and Leader*, but as arrangements for the supply of this part of the Christmas fare had already been made, it was not necessary to take advantage of the offer. The Commanding Officer, Col. J. Griffiths, and Mrs. Griffiths were present during the morning and visited every ward during dinner-time.

In the afternoon various games were indulged in by the men in the various wards, and the "wizard" went round again, this time accomplishing some marvellous performances in the fortune-telling line. A sumptuous tea was provided at four o'clock, when many special dainties were enjoyed through the kindness of many friends of the institution. Mrs. Almeric Paget gave a number of large iced cakes which were distributed among the different wards. After tea some of the patients and nurses engaged in dancing, and impromptu concerts were held in many of the wards.

At 7 p.m. the whole of the sisters and nurses at present on duty at the hospital sat down to their Christmas dinner in the recreation room. Gifts of crackers were received from Mrs. Almeric Paget, and dessert from Miss Foster and Mrs. Griffiths. The Commanding Officer was unable to be present but sent a letter in the course of which he said : "The duties of the nurse are indeed of the noblest order, and it must be a great pride to all who have the opportunity, who possess knowledge, and who can wield their skill to make a man better and even well. At times no human skill, however much backed by knowledge, is of any avail to stem the tide of an overflowing life, but at other times a woman's resources, improved by the best of all that is possible in education and training, gives a help, gentle and almost imperceptible it may be, which suffices to turn the tide from ebb to flow."

### Leicester.

There is no gainsaying the fact that the hospitality of the Leicester hospitals during Christmas has fully equalled their capacity for the reception of wounded soldiers. To explain the analogy the fact must be recalled that in the early days of the war the task was imposed on the military hospital authorities of Leicester of creating accommodation and equipment for 500 beds. This task was only accomplished when the Territorial Association took over the disused County Lunatic Asylum and transformed it into a big military hospital. From that time the provision of hospital accommodation has been continuous. The original hospital has more than doubled its capacity. One hundred and sixty beds have been arranged for in the Leicester Royal Infirmary, the North Evington Poor-Law Hospital has been entirely taken over, convalescent treatment has been provided to the extent of 120 beds in the Gilroes (Corporation Fever) Hospital, fifty-five beds in the Desford Convalescent Home, forty beds in the Knighton V.A.D. Hospital, and other provision made in numerous homes in the county, making the total hospital accommodation about 3,000 beds. Evidence of the largeness of the accommodation, of the central situation of the town, and of the efficiency of the local transport arrangements was forthcoming in the arrival of convoys of sick and wounded on Christmas Eve and on Christmas night. It may be taken for granted that the celebration of Christmas in the hospitals was in keeping with the efficiency of the general hospital arrangements. At the 5th Northern Hospital and at the North Evington Infirmary wards were decorated and Christmas fare was provided, with carol singing and entertainments. At the Knighton V.A.D. Hospital, the Gilroes Hospital, and the Desford Auxiliary Hospital Christmas gifts for every patient, carol singing, and entertainments filled the programmes.

At the Royal Infirmary each patient, military and civil, was the recipient of a Christmas gift or, to be correct, a variety of gifts. All the wards were decorated, the two children's wards had Christmas-trees, electrically illuminated by coloured lamps, special fare, dessert, chocolates, nuts, and for adult males tobacco and cigarettes. As one old daddy expressed himself, "We couldn't ha' been treated kinder nohow, but the drug shop would ha' to be raided." In the afternoon the nurses, each with a different-coloured candle in a candlestick, so far as different colours were obtainable, with doctors and visitors, sang carols in each ward in turn. A gift from the Mayor and Mayoress of four large iced cakes for the military wards, "With the best wishes of the Mayor and Mayoress for a merry Christmas," was much admired and better appreciated. Later in the evening games of all kinds were indulged in. One which gave rise to much hilarity was enacted in one of the military wards, where the convalescent soldiers, each in turn, were sent into a dark room for five minutes, when they were invited to show themselves, close one eye, and thread a needle in five seconds. To ensure that one eye was closed the D.C. found it necessary to cover one eye—to be correct, one side of the face—with his hand, which had previously been well rubbed on a lump of coal, so that after a few tries, in which no one succeeded in threading the needle, the effect of the effort was electrical.

### Liverpool.

1st WESTERN HOSPITAL, FAZAKERLEY.

The 1st Western Hospital was beautifully decorated, the sisters in charge of the wards having vied with each other in their efforts to give the institution a festive appearance. The soldier patients enjoyed the fare provided and had a good time generally. The officers in command paid a visit during the day and were accorded a hearty "cheering" welcome. The staff gave an entertainment in the evening which filled the patients' "cups of happiness" to the full.

Highfield Auxiliary Hospital.

To this hospital is attached a band of lady visitors who contributed unsparingly of time and money to make the wounded soldiers have a happy Christmas. The menu for the mid-day meal consisted of turkey, plum-pudding, fruit, chocolates, and lemonade. Whilst the dinner was in progress Mr. Thomas White, the chairman of the Hospital Committee, entered the beautifully decorated hall where the meal was served, and his appearance was the signal for loud and prolonged cheering. He afterwards addressed a few words to the wounded, and was followed by the vice-chairman, Mr. W. B. Stoddart. Each soldier as he left the hall had a dip into an immense bran-pie, which contained gifts for all. A concert and cinematograph entertainment were provided in the evening, and were thoroughly enjoyed.

ROYAL SOUTHERN HOSPITAL.

In the Royal Southern Hospital each patient on awaking in the morning found a present awaiting. At the bedside for the children there were stockings full of toys and for the men parcels of clothing. Delicacies were provided for those who were unable to partake of the sumptuous dinner which was served to the stronger patients. An excellent concert was given in the evening and was highly appreciated.

MILL ROAD INFIRMARY.

In addition to the Christmas fare in this institution gifts and presents were showered upon the inmates, and seasonable festivities have been in progress throughout the week. A delightful concert was given in the recreation room and was attended by the staff and soldiers.

WESTMINSTER ROAD HOSPITAL.

This hospital was plentifully supplied with good things and was visited during the day by the medical officer in charge, Dr. Moyles, and Major and Mrs. Utting. The wards were tastefully decorated, and the patients had a right royal time.

STANLEY HOSPITAL.

The patients in Stanley Hospital enjoyed an old-fashioned Christmas dinner of roast beef and plum-pudding, and each soldier afterwards received a present most suitable to his needs. A concert and dance were given in the evening and the day was unanimously voted a "Happy Christmas."

### Royal Victoria Infirmary, Newcastle.

CHRISTMAS at the Royal Victoria Infirmary, Newcastle-upon-Tyne, was celebrated in the old-fashioned way, the only variation being that the soldiers and civilians enjoyed rather better fare than in previous years. The wards were decorated rather less with holly than usual, but in some there was a lavish display of festoons of coloured paper. In the morning one of the resident doctors personated Santa Claus and gave a present to every patient in the hospital. Service in the chapel followed, and then at twelve o'clock dinner—turkey and plum-pudding. After dinner tobacco for the men and cakes and oranges for the women. From three to seven o'clock bands of performers of varying degrees of excellence gave entertainments in the wards.

### Norfolk and Norwich Hospital.

THE Norfolk and Norwich Hospital was the scene of various festivities appropriate to the Christmas season, though not upon such a scale as was customary in peace-time. The board of management had purposely refrained from making a Christmas appeal; but as events turned out there was plenty of individual enterprise to fill the gap, for the staff, both honorary and resident, take a generous and by no means coldly professional view of their duties, and there is always a warm and spontaneous co-operation to be had from the public outside. There was, of course, no Christmas-tree, for the hospital children have been taken over by the Jenny Lind Infirmary in accordance with an arrangement made at an early stage of the war, so that the hospital might have all the more room for soldier patients, the number in the hospital on Christmas morning being 150. Sir Alfred Jodrell sent the turkeys for the Christmas dinner. The doctors and surgeons got up a fund among themselves for the supply of various seasonable etceteras; and the sisters and nurses, out of resources in some measure contributed by themselves, displayed a wonderful decorative capacity in the treatment of their several wards. In the afternoon Mrs. Beeching, wife of the Dean of Norwich, gave a concert in one of the wards. Mr. E. W. Everitt, one of the honorary surgical staff, entertained the patients of the King Edward Ward at tea and gave them also an entertainment, which Mr. and Mrs. W. R. Gurley had organised. Mrs. Gurley is the well-known song writer Joan Trevalsa.

SANTA CLAUS AT NEWCASTLE (ROYAL VICTORIA INFIRMARY).

### Torquay.

THE TORBAY HOSPITAL.

THE patients were awakened at 6 A.M. by Christmas carols which the nursing staff sang in the corridors. They then discovered a parcel of presents by the side of their beds, which "Father Christmas" had distributed during the night. At 8 A.M. a service was held in the beautifully decorated Warrington Ward. The decorations throughout the building were undertaken by the soldiers, and consisted entirely of flags of the allied nations. The soldiers seemed to provide the amusements of the day. Those in the Ainslie and Louisa Cary wards formed a coloured minstrel troupe, which was well received. The dinner was of the usual Christmas fare—turkey and plum-pudding—and was much enjoyed, as evidenced by the soldiers fetching the cook to visit the wards, in each of which she was given three cheers as a mark of their appreciation.

WESTERN HOSPITAL FOR PATIENTS OF A CONSUMPTIVE TENDENCY.

The wards in this hospital, which possesses forty beds, were prettily decorated with flags and flowers. The festivities commenced on Christmas Eve with a musical entertainment, after which the Christmas-tree was stripped, each soldier receiving a parcel of good things. The dinner on Christmas Day consisted of the usual turkey and plum-pudding.

## Figures and Tables

**Figure f1:**
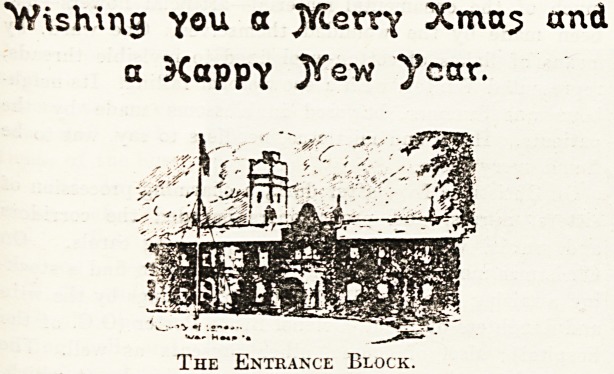


**Figure f2:**
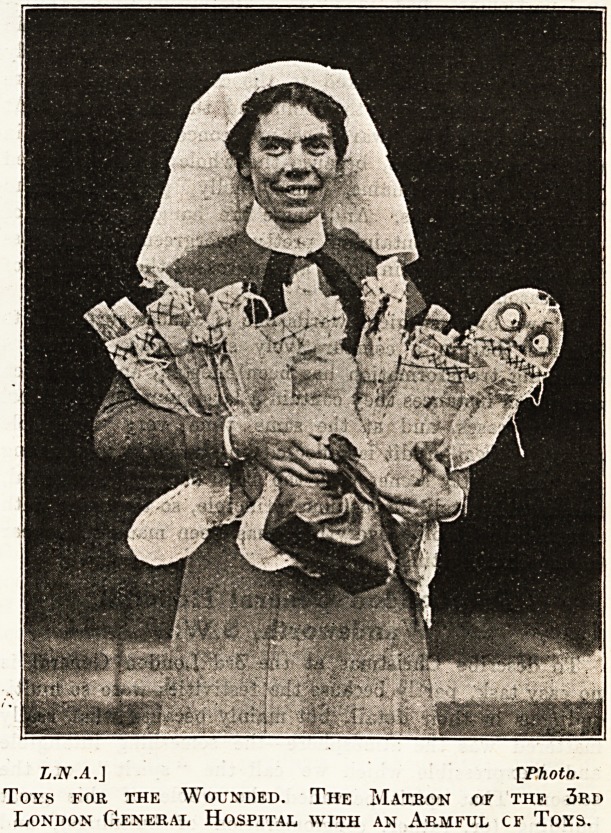


**Figure f3:**
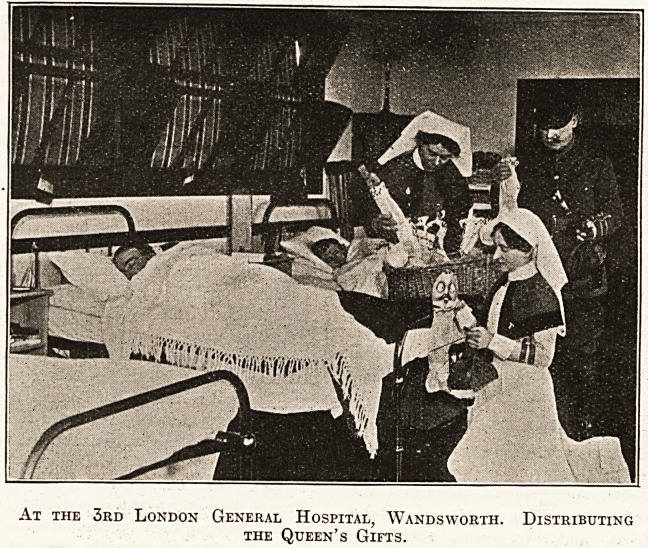


**Figure f4:**
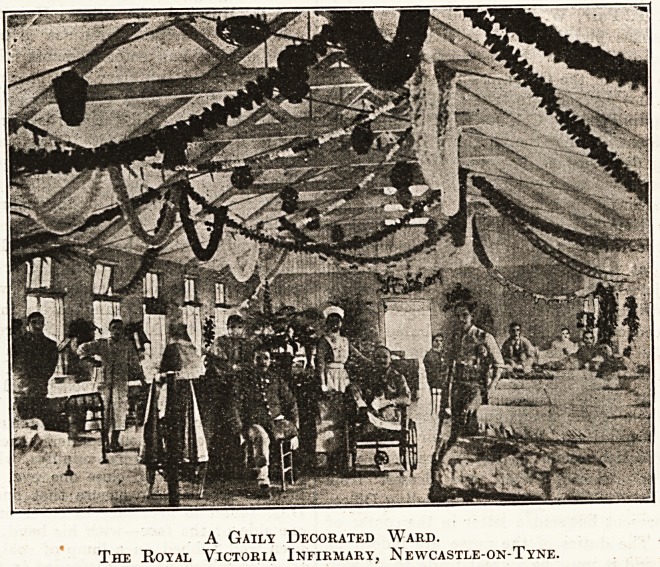


**Figure f5:**