# Errata

**Published:** 1916-01-08

**Authors:** 


### Errata.

WE regret that two errors crept into our correspondent's account of the Christmas festivities at the 3rd London General Hospital. The photograph which we reproduced represented the assistant matron (Miss Northover), not the matron (Miss Holden); and the statement that the patients' "Christmas stockings" were filled by Mrs. Bruce Porter and her daughters was incomplete, the contents of the stockings being kindly supplied by the Ladies' Committee.

